# The antitumoral effects of chemerin are independent from leukocyte recruitment and mediated by inhibition of neoangiogenesis

**DOI:** 10.18632/oncotarget.28056

**Published:** 2021-09-14

**Authors:** Ingrid Dubois-Vedrenne, Diana Al Delbany, Olivier De Henau, Virginie Robert, Maxime Vernimmen, Francina Langa, Anne Lefort, Frédérick Libert, Valérie Wittamer, Marc Parmentier

**Affiliations:** ^1^I.R.I.B.H.M and Welbio, Université Libre de Bruxelles, Campus Erasme, B-1070 Brussels, Belgium; ^2^Centre d'Ingénierie Génétique Murine, Institut Pasteur, 75724 Paris, France; ^3^Present address: Institute for Medical Immunology, Université Libre de Bruxelles, 6041 Gosselies, Belgium; ^4^Present address: iTeos Therapeutics, 6041 Gosselies, Belgium; ^5^Present address: Ambiotis SAS, Canal Biotech 2, 31400 Toulouse, France

**Keywords:** chemerin, ChemR23, CMKLR1, Rarres2, tumor angiogenesis

## Abstract

Chemerin, a multifunctional protein acting through the receptor ChemR23/CMKLR1, is downregulated in various human tumors and was shown to display antitumoral properties in mouse models of cancer. In the present study, we report that bioactive chemerin expression by tumor cells delays the growth of B16 melanoma and Lewis lung carcinoma *in vivo*. A similar delay is observed when chemerin is not expressed by tumor cells but by keratinocytes of the host mice. The protective effect of chemerin is mediated by CMKLR1 and appears unrelated to the recruitment of leukocyte populations. Rather, tumors grown in the presence of chemerin display a much smaller number of blood vessels, hypoxic regions early in their development, and larger necrotic areas. These observations likely explain the slower growth of the tumors. The anti-angiogenic effects of chemerin were confirmed in a bead sprouting assay using human umbilical vein endothelial cells. These results suggest that CMKLR1 agonists might constitute therapeutic molecules inhibiting the neoangiogenesis process in solid tumors.

## INTRODUCTION

Chemerin is a multifunctional protein of 16 kDa, secreted as an inactive precursor by many cell types, including fibroblasts, myocytes, hepatocytes, adipocytes, and many epithelial cells [1–3]. CMKLR1, also named ChemR23 or chemerin_1_, was the first receptor described for chemerin [1, 4]. CMKLR1 belongs to the rhodopsin-like family of G protein-coupled receptors and is coupled to the G_i/o_ family, inhibiting cAMP accumulation and triggering IP_3_-dependent calcium mobilization and activation of the ERK1/2 and PI3K/AKT cascades [5–7]. Chemerin also promotes β-arrestin recruitment and efficient CMKLR1 internalization. Chemerin was initially described as a chemotactic factor for leucocyte populations expressing CMKLR1, which include macrophages, immature myeloid and plasmacytoid dendritic cells (DC), and natural killer cells (NK) [1, 8, 9]. CMKLR1 is also expressed by adipocytes [10, 11], endothelial cells [12], and muscle cells [13].

Besides CMKLR1, chemerin was later shown to bind two other receptors. GPR1, a receptor structurally related to CMKLR1, was described as responding to chemerin in a β-arrestin recruitment assay (TANGO) [14]. Although chemerin promotes very efficiently β-arrestin recruitment to GPR1 and receptor internalization, the signaling ability of GPR1 through the IP_3_/Ca^2+^ and ERK1/2 cascades appears limited [14, 15]. Chemerin_2_ was recently proposed as the new official name of GPR1 [4].

CCRL2 constitutes a third receptor for chemerin. It binds chemerin with high affinity but has so far not been associated with any known signaling cascade, nor to an increase in CCRL2 internalization or turnover [15–17]. CCRL2 is therefore considered as a binding site for chemerin, allowing cells expressing this receptor to display the ligand at their surface and activate neighboring cells expressing CMKLR1 [16].

Chemerin is secreted by cells as an inactive precursor, prochemerin, which is poorly active on its receptors. Various proteases are able to activate prochemerin by removing six or seven amino acids from its C-terminus. These proteases include neutrophil elastase, cathepsin G as well as proteases of the coagulation and fibrinolytic cascades [5–7]. Bioactive chemerin is therefore generated in inflammatory conditions, following tissue injury and during tissue remodeling. Through the recruitment of leukocyte populations and possibly other mechanisms, chemerin plays both pro-and anti-inflammatory roles in various experimental disease models [18–21]. Chemerin was reported to contribute to disease states by promoting inflammation in autoimmune encephalomyelitis [22], chronic obstructive pulmonary disease [21], and psoriasis [2], but to dampen inflammation in viral and aseptic lung disease models [3, 19, 23], atherosclerosis [24] and pancreatitis [25].

Chemerin was described as an adipokine secreted by adipocytes, regulating adipocyte differentiation and insulin sensitivity of adipocytes and skeletal muscle cells [11, 26], although the importance of chemerin as a regulator of energetic metabolism is a matter of debate [27]. Chemerin blood levels have been correlated in many reports to obesity and parameters of the metabolic syndrome, as well as to many inflammatory conditions. However, the assays used in most of these studies do not discriminate between active and inactive forms of chemerin, and the functional significance of these observations is often questionable. A recent study suggested that human obesity is indeed associated with increased systemic (pro)chemerin levels but not accompanied by higher chemerin bioactivity [28].

Chemerin was reported to promote the formation of capillary-like structures in a co-culture model and to increase proliferation, migration, and tubulogenesis of endothelial cells, as well as the release of matrix metalloproteinases (MMP) [12, 29]. A positive effect of chemerin on angiogenesis was also reported *in vivo* in a Matrigel plug assay and the mouse corneal angiogenesis model. The role of the ERK1/2 and PI3K/AKT pathways was proposed in this context [30]. Chemerin was also reported to promote ERK activation in decidual endothelial cells and the formation of capillary-like tube structures, suggesting a role in vascular remodeling during early pregnancy [31].

Provided its roles in leukocyte recruitment, angiogenesis, and regulation of metabolism, chemerin has potentially diverse possible actions on the tumor microenvironment, and a growing number of reports have highlighted the role of chemerin in cancer. Both pro- and anti-tumoral effects of chemerin have been described according to the type of cancer considered [32–34]. Chemerin levels were found elevated in the blood of patients with many different types of cancer [35]. Expression in the tumor itself was most often turned down compared with normal tissue in many cancer types, including breast, lung, and prostate cancers, adrenocortical and hepatocellular carcinomas, melanoma, and squamous cell carcinoma of the skin. However, increased levels of chemerin were reported in some cancer types, such as squamous cell carcinoma of the oesophagus, gastric cancer, mesothelioma, and neuroblastoma [34]. Several mechanisms have been proposed to explain the pro- or anti-tumoral properties of chemerin. Pro-tumoral properties are attributed to a direct effect of chemerin on tumor cells, thereby stimulating their proliferation and migration [34]. The anti-tumoral effects of chemerin in a mouse model of melanoma were attributed to the recruitment of effector NK cells [36]. Chemerin was also described to inhibit the metastatic process of hepatocellular carcinoma by inhibiting the PI3K/AKT pathway through the release of PTEN from a complex with CMKLR1 [37]. Transcriptional upregulation of PTEN and downregulation of PD-L1 were reported as a consequence of chemerin action on human prostate cancer and sarcoma cell lines [38]. A direct effect on tumor cells was also proposed to explain the inhibition of growth and bone invasion by breast cancer cells [39].

In the present study, we investigated the role of chemerin in two tumor graft models in mice. We observed that bioactive chemerin overexpression displayed antitumoral properties independently from its expression site, properties entirely mediated through CMKLR1. These effects did not involve the recruitment of leukocyte populations to the tumors. It was rather found that chemerin prevents efficient angiogenesis in growing tumors, resulting in hypoxia and an increase in necrotic cell death. Contrasting with previously reported data, we were unable to detect proangiogenic properties of chemerin in various assays but report rather strong anti-angiogenic effects in a bead sprouting assay.

## RESULTS

### Expression of chemerin by tumoral cell lines delays tumor growth

We tested the effect of chemerin in two models of syngeneic tumor grafts, involving the B16 melanoma and the Lewis lung carcinoma (LLC) cell lines. Both tumoral cell lines are derived from the C57BL/6 strain of mice. As determined by qRT-PCR, these cell lines in culture do not express significant levels of chemerin, nor CMKLR1 and GPR1, the two functional chemerin receptors (data not shown). B16 and LLC cells were transfected with a bicistronic plasmid (pEFIN5), encoding a pre-processed form of chemerin (1-157), not requiring additional C-terminal clipping to display bioactivity, or the empty vector. Following selection by G418, individual clones were collected. The production of bioactive chemerin was tested by an aequorin-based calcium-mobilization assay using a CHO-K1 cell line stably expressing CMKLR1. Conditioned media from cells expressing chemerin(1-157) stimulated calcium release in CMKLR1-expressing cells, while that of cells transfected with the empty vector did not (Supplementary Figure 1A). The concentration of bioactive chemerin in the conditioned medium of B16 and LLC cells was estimated respectively to 1.8 and 2.2 nM. Chemerin at 100 nM did not affect the growth of B16 and LLC cells in culture, and the expression of chemerin by the cells themselves did not affect the growth of the recombinant cell lines (Supplementary Figure 1B and 1C).

**Figure 1:**
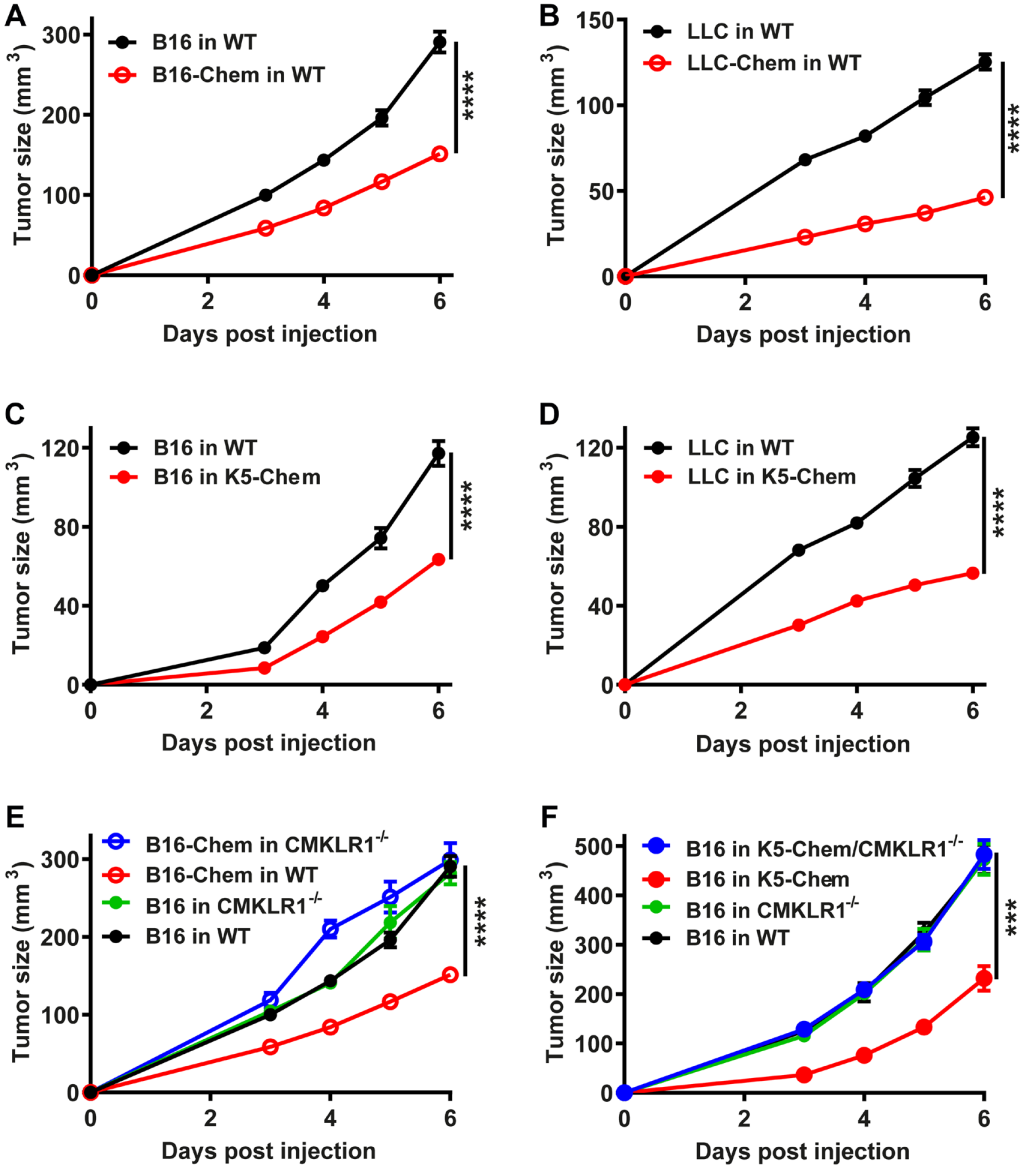
Anti-tumoral properties of the chemerin-CMKLR1 system. B16 (**A**) or LLC (**B**) cells (1.10^6^ per mouse) expressing or not bioactive chemerin were grafted subcutaneously into WT mice. B16 (**C**) or LLC (**D**) cells were grafted subcutaneously into WT or K5-chemerin mice. (**E**) Control and CMKLR1^−/−^ mice were grafted with B16 cells expressing or not chemerin. (**F**) Control, K5-chemerin, CMKLR1^−/−^ and K5-chemerin/CMKLR1^−/−^ mice were grafted with B16 cells. The tumor size was measured daily from day 3. The results (mean ± SEM) are the compilation of three independent experiments with 5 mice or more per group in each experiment. The statistical difference (^***^ *P* < 0.001 and ^****^ *P* < 0.0001) was estimated by the Mann-Withney test.

The cell lines were grafted subcutaneously in the back of syngeneic C57BL/6 mice. When B16 melanoma cells expressing bioactive chemerin were grafted, the tumors grew significantly slower than following the graft of untransfected B16 cells or cells transfected with the vector alone (Figure 1A). These results are in agreement with a previous report [36]. When LLC cells were grafted, bioactive chemerin expression by the cells also reduced significantly the growth of the tumors, the delay being more pronounced than for B16 cells (Figure 1B). Together, these findings showed that the expression of bioactive forms of chemerin by different tumoral cell lines decreases considerably the growth of tumors *in vivo*. Various leukocyte populations involved in the host response to cancer (macrophages, dendritic cells, NK cells) express CMKLR1. A potential mechanism explaining the anti-tumoral effect of chemerin was, therefore, the recruitment of leukocytes, and this hypothesis was supported by a previous report describing the role of NK cells [36].

### Overexpression of bioactive chemerin by host skin keratinocytes also delays tumor growth

To evaluate whether the site of expression of chemerin is of relevance for its anti-tumoral effects, we used a mouse model in which a bioactive form of chemerin is expressed under control of the keratin 5 promoter (K5-chemerin model). These mice were previously shown to express chemerin in basal keratinocytes of the skin, resulting in a significant increase of chemerin activity in the blood [40]. Following injection of B16 (Figure 1C) or LLC (Figure 1D) cells, K5-chemerin mice developed tumors significantly smaller than control mice in both tumor graft models. The growth delay was similar to that observed when chemerin is expressed by the tumor cells themselves. These results indicate that the consequences of chemerin expression on tumor growth are independent of where the protein is produced, which suggests that a gradient driving the recruitment of leukocyte populations toward the tumor is not required.

### CMKLR1 mediates the anti-tumoral effects of chemerin

CMKLR1 is a fully functional chemerin receptor, while GPR1 displays only weak signaling properties [15]. To determine whether CMKLR1 is involved in the protective effects of chemerin, we tested the consequences of chemerin overexpression on the tumor graft models, using mice invalidated for CMKLR1. In the B16 graft model, CMKLR1 invalidation had no influence by itself on the tumor growth but abrogated completely the effect of chemerin expression by the tumor cells (Figure 1E). Similarly, expression of chemerin by basal keratinocytes in K5-chemerin mice did not affect the growth of B16 tumors when these mice were also deficient for CMKLR1 (Figure 1F), and the growth curves were similar to those of B16 cells in WT mice. Altogether, we conclude that the anti-tumoral properties of chemerin in these tumor graft models are entirely mediated by CMKLR1.

### The anti-tumoral effects of chemerin appear independent from leukocyte recruitment

**Figure 2:**
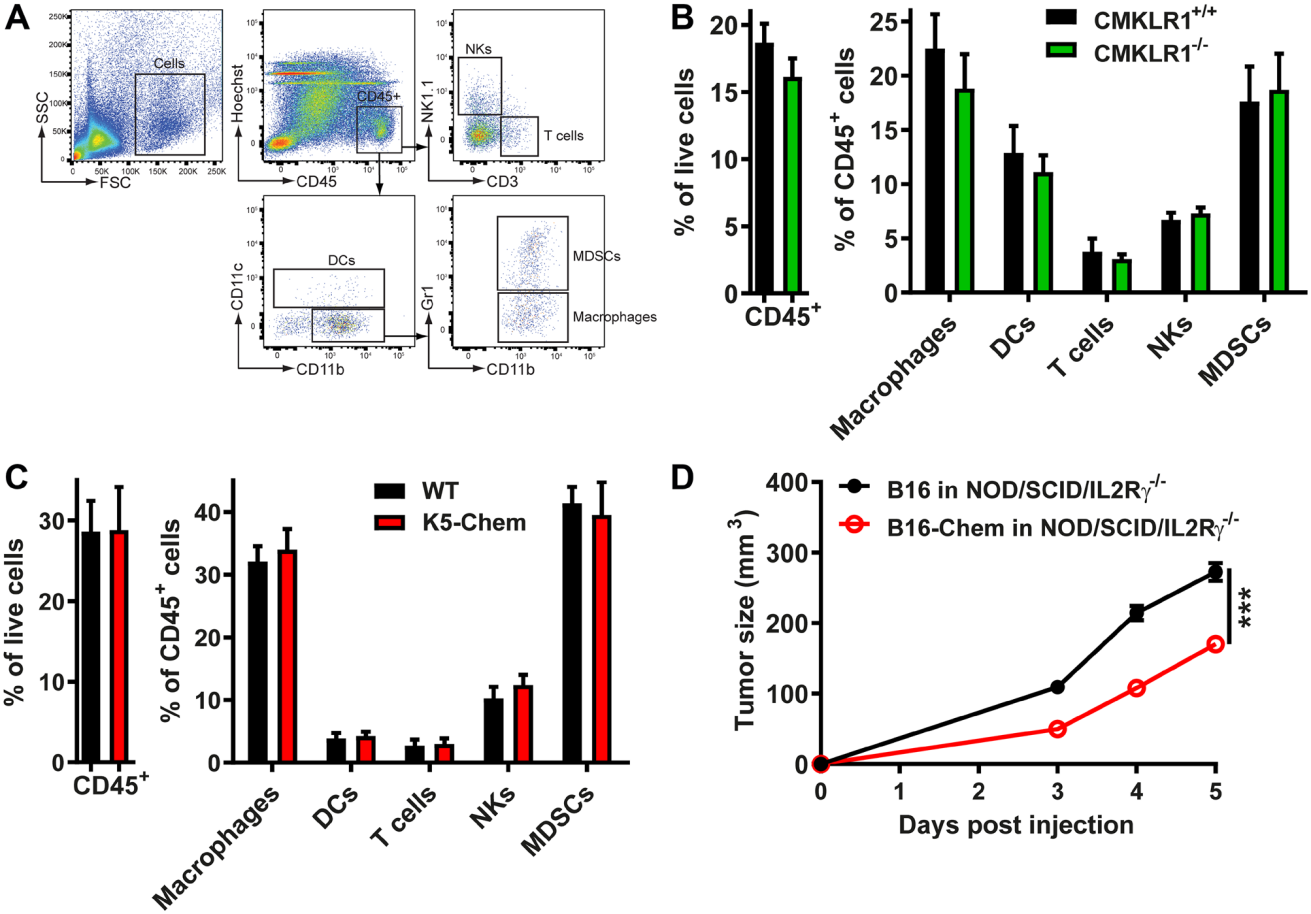
The anti-tumoral effect of chemerin is unrelated to leukocyte recruitment. (**A**) Flow cytometric gating used to quantify leukocyte subsets in a representative sample. (**B** and **C**) B16 cells were grafted to control and CMKLR1^−/−^ mice (B) or to control and K5-chemerin mice (C). Six days after the graft, the tumors were collected and digested, the cell suspension stained with combinations of antibodies, and analyzed by flow cytometry. The percentage of CD45^+^ cells and the proportion of various leukocyte subsets (% of CD45^+^ cells) recruited to the tumors are represented, including T cells (CD3^+^ NK1.1^−^), macrophages (CD11b^+^ CD11c^−^ Gr1^−^), DCs (CD11c^+^), NK cells (CD3^−^ NK1.1^+^) and MDSCs (CD11b^+^ CD11c^−^ Gr1^+^). The data combine three independent experiments with 5 mice per condition in each experiment. (**D**) B16 cells expressing or not chemerin were grafted to NOD/SCID/IL2Rγ^−/−^ mice and the tumor size monitored daily. The results are from a representative experiment out of two performed (mean ± SEM, *n* = 6 mice per group, ^***^ *P* < 0.001, Mann-Withney test).

Various leukocyte populations affect tumor development. Cytotoxic T cells, M1 macrophages, and NK cells contribute to anti-cancer defenses, while M2 macrophages, regulatory T cells, myeloid-derived suppressor cells (MDSCs), and tolerogenic dendritic cell subsets may contribute to tumor progression [41]. We therefore tested whether the chemerin/ CMKLR1 system might influence tumor growth and progression by affecting the set of leukocytes recruited to the tumor microenvironment. Leukocyte populations in B16 tumors were analyzed by FACS following enzymatic digestion of the tissue. On day 5 post-injection, the number of CD45^+^ cells and the proportions of different leukocyte subpopulations such as T cells, macrophages, DCs, NK cells, and MDSCs were similar in tumors from WT or CMKLR1^−/−^ mice (Figure 2A and 2B). We also compared B16 tumors from WT and K5-chemerin mice (Figure 2C). LLC or B16 cells expressing or not chemerin and different time points after the tumor graft were also investigated (data not shown). No significant change in the recruitment of a specific leukocyte subset was observed in any of these situations. These data suggested that chemerin expressed by the tumor cells or by the host acts on tumor development independently of the recruitment of leukocytes. In order to confirm this conclusion, B16 cells expressing or not chemerin were grafted to NOD/SCID/IL2Rγ^−/−^ immunodeficient mice. As in WT mice, the tumors expressing chemerin were significantly smaller (Figure 2D), confirming that the immune system is not involved in the effects of chemerin on tumor growth.

### Tumoral angiogenesis is impaired in the presence of chemerin

**Figure 3:**
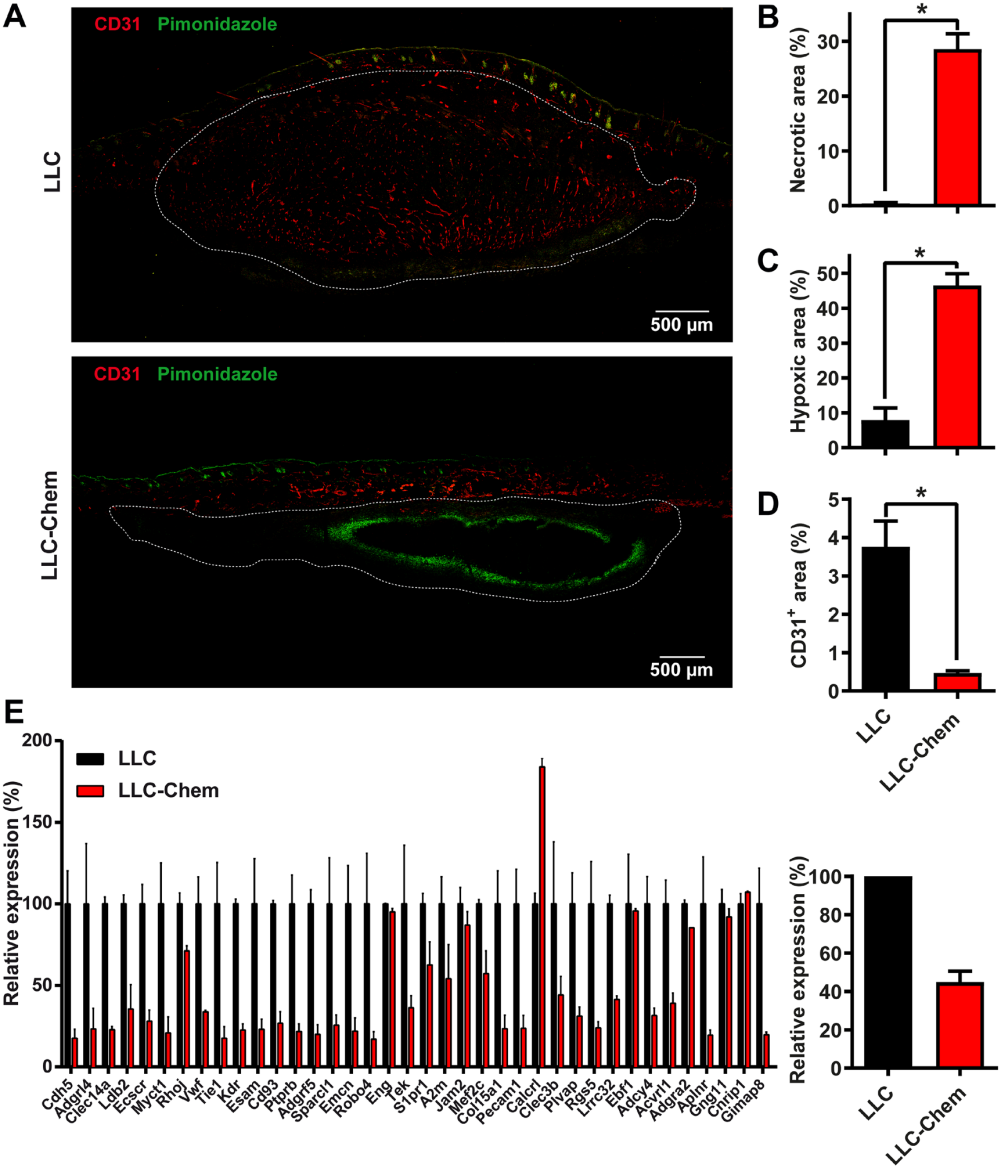
Chemerin expression promotes hypoxia and necrosis in tumors by decreasing angiogenesis. (**A**) Immunostaining of CD31 and pimonidazole in LLC tumors expressing or not chemerin at day 5 after the graft. (**B**, **C**, and **D**) The relative area of necrosis (B), hypoxia (C, pimonidazole^+^), and endothelial cells (D, CD31^+^) was measured on cryosections following staining with anti-pimonidazole and anti-CD31 antibodies. The results are from a representative experiment out of 3 performed (mean ± SEM, *n* ≥ 4 mice per group, ^*^ *P* < 0.05, Mann-Withney test). (**E**) Relative expression of the first 38 genes of an angiogenesis signature (described for human cancer types [42]) in LLC tumors expressing or not chemerin (left panel). The average expression level for all 38 genes is represented in the right panel. These data were extracted from RNAseq experiments. For each condition, two pools of 3 tumors, collected 5 days after the graft, were analyzed. The data (mean ± SEM) were normalized relative to WT-LLC tumors (100%).

Angiogenesis also plays a major role in tumor development, particularly in the late stages of progression in which chemerin was shown to act [40]. Histological analysis of the LLC and B16 tumors at different stages of growth uncovered the presence of necrotic areas predominating in the central parts of the tumors and increasing in size with time. Although variable from one tumor to another, the average surface of necrosis was significantly larger in tumors expressing chemerin than in control tumors, and in tumors grown in K5-chemerin mice than in tumors from WT mice, despite their smaller size (Figure 3A and 3B and data not shown). Hypoxia was evaluated in tumors at an early time point (5 days post-grafting for LLC tumors) by intravenous injection of pimonidazole 90 min before euthanasia. Hypoxic regions were quantified on cryosections following staining with a FITC-conjugated anti-pimonidazole antibody. The surface of hypoxia was much larger in LLC tumors expressing chemerin (Figure 3A and 3C). We also quantified endothelial cells in tumors after labeling with an anti-CD31 antibody. The relative surface of CD31^+^ staining was significantly smaller in LLC mice expressing chemerin (Figure 3A and 3D). Altogether, these data suggested impaired angiogenesis in tumors growing in a context of bioactive chemerin overexpression.

We next performed an RNAseq profile of LLC tumors (5 days following graft) expressing or not chemerin. When considering the first 1000 upregulated genes in chemerin-expressing tumors (with FDR < 0.007), the most upregulated signature in the hallmark gene sets (GSEA database) was hypoxia (*p* = 2.8 10^−18^). Out of the 200 genes included in this gene set, 29 were significantly upregulated in chemerin-expressing tumors (BHLHE40, F3, CITED2, SDC2, CAV1, CDKN1B, PRKCA, VEGFA, ENO2, TIPARP, PNRC1, HK2, P4HA1, EGFR, MXI1, ERO1L, STC1, AK4, ZNF292, GYS1, NDRG1, ADM, BNIP3L, PDK1, GPI, CCNG2, MT2A, HOXB9 and KDM3A). We also observed in these tumors a clear downregulation of an angiogenesis signature (Figure 3E and Supplementary Figure 2) described for a set of human cancer types [42].

**Figure 4:**
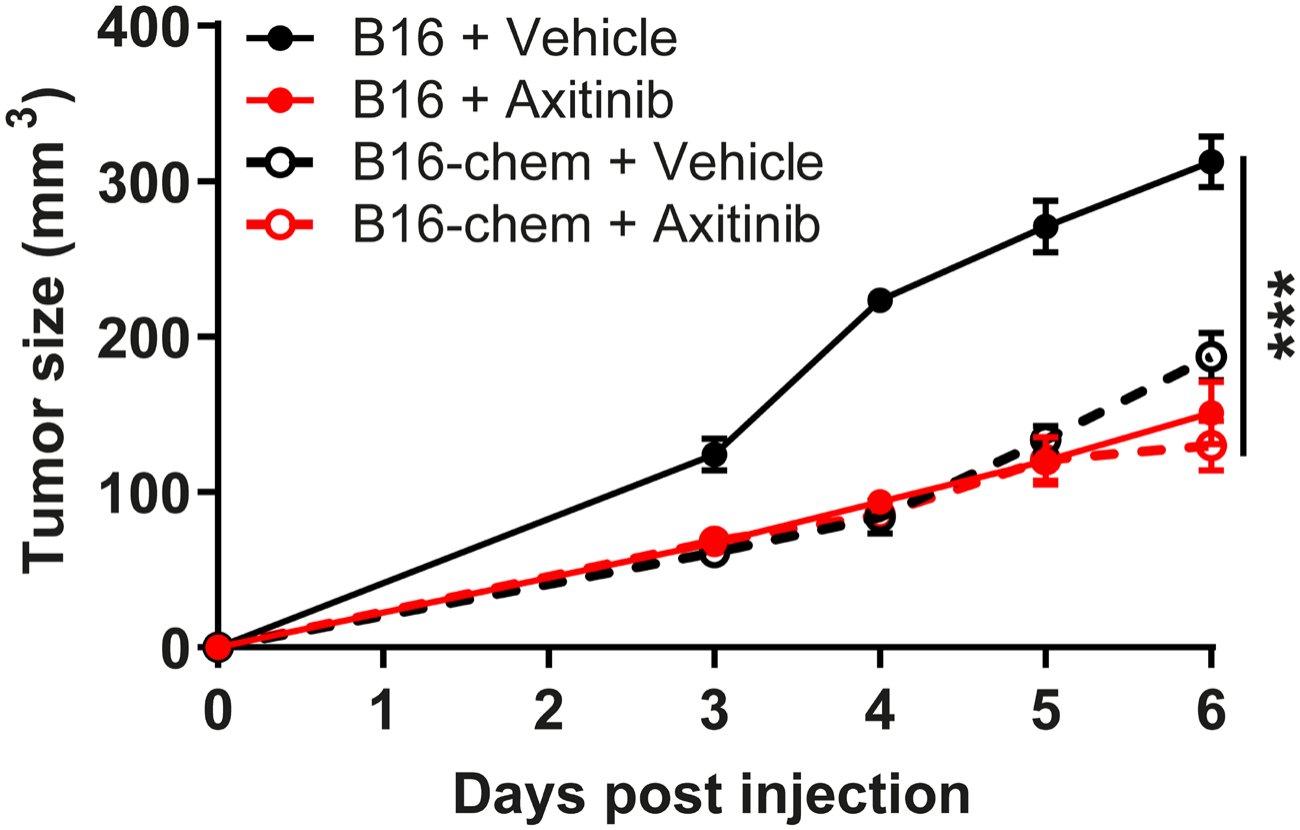
Chemerin does not cooperate with axitinib. Mice were grafted with B16 cells expressing or not chemerin and treated by gavage with axitinib or the vehicle. The size of the resulting tumors was measured daily from day 3. The results are from a representative experiment out of two performed (mean ± SEM, *n* = 5 mice per group, ^***^ *P* < 0.001, Mann-Withney test).

Provided the anti-angiogenic and anti-tumoral properties of chemerin in the tumor models, we compared the consequences of chemerin overexpression with that of an anti-angiogenic agent used to treat various human cancer types, the vascular endothelial growth factor receptor (VEGFR) tyrosine kinase inhibitor axitinib. The growth of B16 tumors was similarly impaired in chemerin-overexpressing mice and in control mice treated daily with 10 mg/kg axitinib (Figure 4), demonstrating that chemerin displays, in this model, properties as potent as a reference compound used in the clinic. The combination of chemerin overexpression and axitinib did not delay further the progression of B16 tumors, suggesting the absence of cooperativity. The histology of tumors expressing chemerin and/or grown in mice treated with axitinib did not exhibit significant differences in terms of necrosis or density of blood vessels.

### Chemerin overexpression does not increase vascular permeability

CMKLR1 is expressed by endothelial cells [12], and the tumor angiogenesis is impaired in the context of bioactive chemerin overexpression. The permeability of vessels was therefore assessed in K5-chemerin and CMKLR1^−/−^ mice by the Evans Blue extravasation assay, both in basal conditions and in the context of a local inflammatory state promoted by TPA (Supplementary Figure 3). As expected, vessel permeability was increased in all mice after TPA treatment, but the overexpression of chemerin or the absence of CMKLR1 did not modify the amount of dye extracted from tissues, either in basal or inflammatory conditions. These data suggest that the overexpression of bioactive chemerin or the absence of CMKRL1 does not significantly affect the permeability of the endothelium in normal tissues.

### Chemerin inhibits angiogenesis in the bead sprouting assay

**Figure 5:**
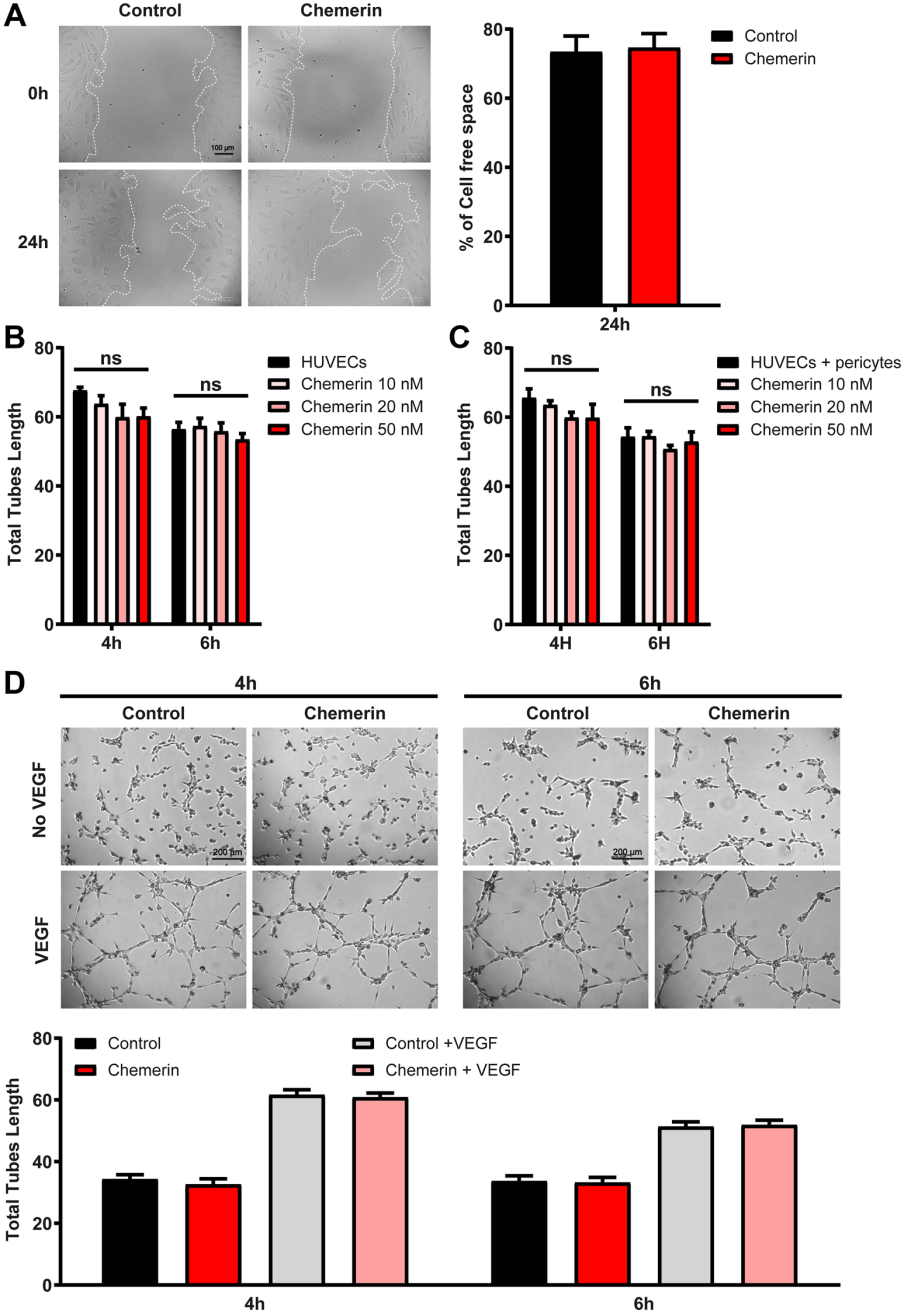
Effect of chemerin on *ex vivo* angiogenesis assays. (**A**) Wound healing assay on HUVECs in the presence or not of 5 nM recombinant human chemerin. Images were captured immediately after removal of the insert and 24 h later (left panel). The cell-free area relative to that at T_0_ (%) was calculated (right panel). (**B**, **C**, and **D**) The formation of tubes by HUVECs in monocultures (B) or co-cultures with pericytes (C) was measured in the presence of VEGF (#CC-4114A) and different concentrations of chemerin (10 nM, 20 nM, or 50 nM). (D) Representative images (top panel) and measurement of tube length (bottom panel) in HUVECs cultures in the presence of VEGF and/or 20 nM human chemerin after 4 and 6 hours. The data are from 3 independent experiments with 3 wells per condition in each experiment (mean ± SEM, Mann-Withney test).

**Figure 6:**
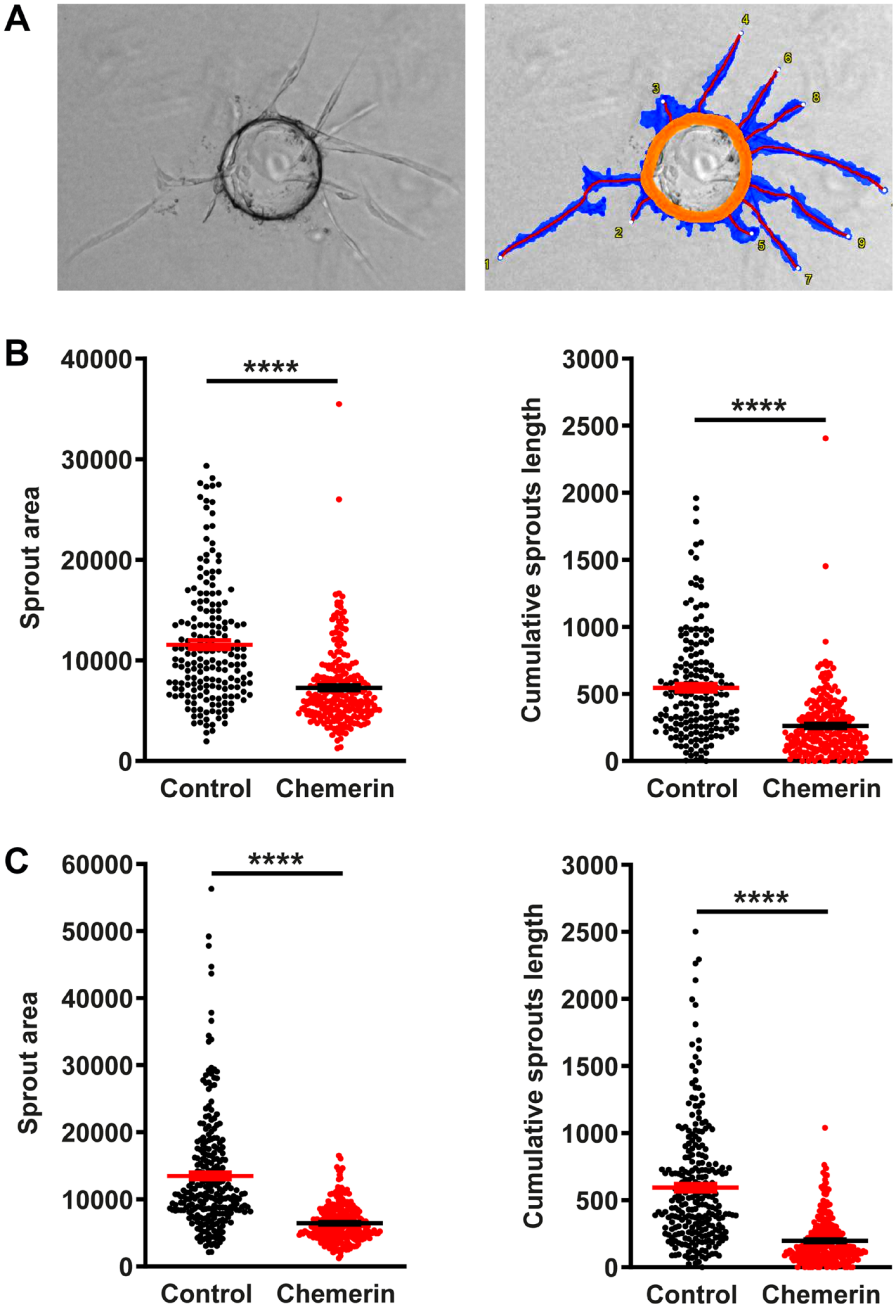
Chemerin inhibits angiogenesis in the bead sprouting assay. (**A**) Illustrative image of a bead bearing sprouts and its analysis by the Wimasis software. The orange circle identifies the bead, the blue area the surface covered by sprouts, and the red lines the length of the sprouts. (**B**) The sprouts area and the cumulative sprouts length were measured for beads grown in medium containing (red squares, *n* = 248 beads) or not (black circles, *n* = 186 beads) chemerin at the concentration of 5 nM, at days 7 after seeding. (**C**) Same experiment at day 10 after seeding (*n* = 256 beads for each condition). The data (mean ± SEM) are from 2 independent experiments with 8 wells per condition in each experiment. The statistical difference (^****^ *P* < 0.0001) was estimated by the Mann-Withney test.

Chemerin was previously reported to promote angiogenesis in the tube formation assay using human umbilical vein endothelial cells (HUVEC) [12, 29]. We investigated the effect of human chemerin on HUVECs in several settings. In a wound-healing assay, chemerin did not modify the closing of the wound either positively or negatively (Figure 5A). Similarly, in our hands, chemerin did not significantly affect the formation of tubes by HUVECs grown on Matrigel (Figure 5B) in the presence of VEGF, at any of the concentrations tested (10-20-50 nM). In the presence of pericytes in the tube formation assay, chemerin addition had again no significant effect on the outcome (Figure 5C). In the absence of VEGF, chemerin was also unable to modify the organization of endothelial cells into tubes (Figure 5D). However, in a tridimensional model of angiogenesis, the bead sprouting assay, chemerin strongly inhibited the angiogenesis process by decreasing the number, total length, and total surface of sprouts (Figure 6). The inhibitory effect was more pronounced when sprouting initiation was allowed for 5 days in the presence of high concentrations of growth factors, and their concentrations were reduced by half until analysis at days 7 and 10. These data support that chemerin affects the growth or stability of endothelial cell sprouts in a model mimicking closely the way neovessels form *in vivo*.

## DISCUSSION

Chemerin is a pleiotropic factor acting as a chemoattractant factor for leukocyte populations but also regulating vascular tone, glucose, and lipid metabolism as well as reproductive functions [20]. Correlation studies in human have highlighted a decrease of chemerin expression in several solid tumors, including adrenal, lung, and prostate carcinoma, as well as melanoma. Several mouse models of cancer have also supported an anti-tumoral role of chemerin, including in the B16 melanoma model [36]. We confirmed here that B16 melanoma cells producing bioactive chemerin grow less efficiently than wild-type B16 cells. Similar observations were made with Lewis lung carcinoma cells expressing chemerin, confirming the protective role of chemerin in another model.

CMKLR1 is the only chemerin receptor endowed with a full signaling repertoire, including G protein- and arrestin-mediated pathways. It is expressed in various cell populations that contribute to the tumor micro-environment and may influence positively or negatively tumor progression. CMKLR1 expression was also described in endothelial cells, suggesting another mechanism by which chemerin may control tumor development. It is expected that an effect of chemerin mediated by the recruitment of a leukocyte subset would require the presence of a chemerin gradient attracting the cells, and would therefore be dependent on the expression of chemerin by the tumor itself. To investigate whether this hypothesis holds true, we used our previously described transgenic model in which a bioactive form of chemerin is expressed under the control of the keratin K5 promoter [40]. In contrast to prochemerin, which is constitutively produced by many cell types, the chemerin expressed in this transgenic model does not require proteolytic processing, as the codons encoding the last 6 amino acids of prochemerin have been removed from the transgene construct. Active chemerin is therefore produced by basal keratinocytes, and both chemerin immunoreactivity and bioactivity are significantly increased in the blood flow [40]. Grafting B16 and LLC cell lines in these mice resulted in a slower growth rate, similar to what was observed when chemerin is expressed by the tumor cells themselves. It appeared therefore that the site of chemerin expression is not important for its anti-tumoral effects. If chemerin gradients were still present in these mice, they should drive CMKLR1-expressing cells to the skin rather than to the tumor graft. These results question the contribution of leukocyte recruitment in the anti-tumoral effects of chemerin.

As the main functional receptor of chemerin, it was postulated that CMKLR1 mediates the effects of chemerin on tumorigenesis, and we tested the effect of chemerin in the context of mice invalidated for this receptor. We observed that in the B16 syngeneic graft model, the absence of CMKLR1 reversed completely the effect of chemerin, whether chemerin is produced by the tumor cells or by the host keratinocytes. These observations demonstrate the implication of CMKLR1 in the process. In accordance with other studies [36], CMKLR1 invalidation did not modify the growth of B16 or LLC syngeneic grafts. We reported previously that, in a chemical model of skin carcinogenesis, CMKLR1 deficiency had similarly no consequences, but that CMKLR1 KO mice developed spontaneous tumors of the skin [40]. In different cancer models, it appears therefore that the endogenous production of chemerin does not result in a significant anti-tumoral effect. The B16 and LLC graft models bypass however the slow progression of naturally occurring tumors, and the fact that CMKLR1-deficient mice display a high frequency of spontaneous skin tumors suggests that the endogenous chemerin production may indeed counteract tumor development in natural situations. The various reports showing down-regulation of chemerin expression in different cancers [36, 43–45] suggest that such anti-tumoral effect of endogenous chemerin may occur in human as well.

In a previous study, Pachinsky et al. described an anti-tumoral role of chemerin and attributed this effect to the recruitment of NK cells [36]. Chemerin is well established as a chemotactic factor for NK cells, as well as other leucocyte populations (macrophages, dendritic cells) that may influence tumoral immunity [1, 9, 46, 47]. As stated above, the anti-tumoral effect of chemerin appeared however independent from the expression site. While expression by LLC or B16 tumor cells might favor the recruitment of a specific cell population, such as NK cells, to the tumor, this would not be expected when bioactive chemerin is expressed by the basal skin keratinocytes of the host. Overexpression of chemoattractant factors can however prevent the formation of local gradients necessary for directional leukocyte migration [48], and blood bioactive chemerin levels reach, in K5-chemerin mice, values (0.4 nM) [40] that might indeed compromise the establishment of functional local chemerin gradients. Nevertheless, whether analyzed by immunofluorescence or FACS, the leukocyte populations infiltrating the LLC and B16 tumors were found to be unaffected by the expression of bioactive chemerin by the tumor cells or by the host. The role of leukocytes was further excluded by the similar growth rate observed for B16 tumors expressing or not chemerin grafted in the severely immunodeficient mouse model NSG (NOD-SCID-IL2Rγ KO) that lacks mature T, B and NK cells, and are defective in the function of macrophages and dendritic cells [49]. Altogether, these results demonstrate that the leukocyte subsets found in the tumors of our mouse models are recruited essentially by other factors than chemerin, and that alteration of this system by chemerin overexpression, either in the tumor or the host, does not significantly affect this recruitment. In a model of adrenocortical carcinoma, the anti-tumoral effect of chemerin was also proposed to be independent of leukocyte recruitment [50].

Provided the lack of leukocyte contribution, we considered angiogenesis as another key element in tumor progression that plays an essential role at similar time frames. Tumor progression is indeed highly dependent on neoangiogenesis, as the limit for diffusion of oxygen and nutrients in tissues is in the range of a few hundred μm. In the absence of efficient vascularization, the proliferation of tumor cells is balanced by apoptosis and necrosis driven by hypoxia [51]. We showed that in the presence of high levels of bioactive chemerin, whether produced by tumor cells or the host, the areas of hypoxic and necrotic regions in the tumors are much larger than in control tumors, suggesting inefficient vascularization. As increased hypoxia and necrosis were observed at early time points following the graft of tumoral cell lines, a causal role in the delayed growth of the tumors is likely. We tested further the consequences of chemerin overexpression on the vascularization of tumors. Labeling of endothelial cells by an anti-CD31 antibody in LLC tumors confirmed the lower density of blood vessels in the context of chemerin overproduction. Transcriptome analysis of the tumors confirmed a hypoxic signature, as well as a strong downregulation of an angiogenic signature in tumors grown in the context of chemerin overexpression.

Neovascularization is a multi-step process. Local hypoxia promotes the production of angiogenic factors such as VEGF and FGF-2, leading to the activation of endothelial cells. The digestion of basement membranes by proteases and the disruption of their contacts with pericytes allow endothelial cell proliferation and their chemotaxis by a collective migration process. Following their formation, the neovessels acquire a mature and stable phenotype by restoration of the basal membrane and coverage by pericytes [52]. Many factors contribute to the control of angiogenesis, including leukocyte chemoattractant molecules. A set of chemokines, including CXCL1, CXCL2, and CXCL12, are described as proangiogenic and stimulate endothelial cell chemotaxis, particularly in a tumoral context, whereas CXCL4, CXCL9, and CXCL10 possess angiostatic properties [53, 54]. The role of chemerin in the angiogenesis process has been addressed in a few studies. Chemerin was described to promote the formation of tubes in cultures of human microvascular endothelial cells [12] or in co-cultures of endothelial cells and dermal fibroblasts [29], an effect involving the PI3K and MAPK pathways. In addition, proangiogenic properties of chemerin have also been reported in the corneal model, and Matrigel plugs in mice *in vivo*, as well as in the rat aortic ring assay *ex vivo* [30]. To date however, no studies have directly addressed the link between chemerin and angiogenesis in a tumoral context.

CMKLR1 and CCRL2 expression was described in endothelial cells, and CCRL2 is strongly upregulated in these cells following stimulation by inflammatory cytokines [55]. Transcriptome analyses on mouse pericytes available in databases (GEO datasets GSE64510 and GSE75668) [56] identified that CMKLR1 and CCRL2 are significantly expressed by pericytes as well. Chemerin can therefore act through CMKLR1 on both endothelial cells and pericytes, affecting thereby the formation and stability of neovessels, while CCRL2 might influence the efficacy of chemerin by presenting the molecule to its receptor.

When compared to the effect of axitinib used at a classical dosage in mice, chemerin overexpression appeared as efficient as the VEGFR tyrosine kinase inhibitor. No cooperativity was observed between the two molecules. This observation may indicate that inhibition of VEGFR signaling and chemerin activity converge toward a common pathway. Alternatively, the effect of each molecule individually reaches a maximal efficacy on the angiogenesis process, thereby preventing cumulative outcomes.

The anti-angiogenic effects of chemerin in this tumoral context appear to contradict the pro-angiogenesis properties described earlier. However, in the study by Bozaoglu et al. [29], the activity was observed for concentrations of chemerin of 0.3 to 3 ng/mL (20 to 200 pM), while the proangiogenic effects were lost at a concentration of 10 ng/mL (600 pM). As the EC_50_ of chemerin on CMKLR1 is about 300 pM, the effects of chemerin on angiogenesis were observed only at suboptimal doses. In addition, we have been unable to reproduce the proangiogenic effects of chemerin in the tube formation assay, using HUVECs, as well as in the mouse aortic ring assay (data not shown). We could however observe a strong inhibitory effect in a 3-dimensional angiogenesis assay, the bead sprouting assay. In this setting, HUVECs are seeded on Cytodex beads and placed in a fibrin matrix. Vascular sprouts form in the presence of proangiogenic growth factors, in a process mimicking closely the way neovessels are formed *in vivo* as a result of hypoxia. The time frame (days) is also much longer than that of the tube formation assay (hours). Chemerin reduced the number and size of the sprouts, particularly when the concentrations of growth factors were reduced during the second part of the assay. Future work will be necessary to reconcile the conflicting results dealing with the pro-and anti-angiogenic effects of chemerin *in vivo* and *in vitro*.

In our K5-chemerin model, bioactive chemerin overexpression does not seem to affect the vasculature in normal tissues, which might lead to severe consequences. Tumoral angiogenesis differs significantly from the normal process. It is dominated by the high production of VEGF and other vascular growth factors, leading to the formation of a rather abundant network of neovessels, but these often lack the maturation steps necessary to achieve efficient blood supply. The vascular bed of solid tumors often presents structural abnormalities, with tortuous vessels, haphazard patterns of interconnection and branching, and dilated segments. Also, tumoral vessels are often leaky as a result of incomplete coverage by pericytes [57]. The tumoral vasculature, which remains permanently immature, may therefore be much more sensitive to the destabilization effects of chemerin than normal vessels. Such a specific effect on tumoral angiogenesis might constitute an advantage in terms of therapeutic applications. Future will tell whether targeting the chemerin-CMKLR1 system with agonists, thereby affecting the formation and/or stabilization of neovessels, may constitute an additional and complementary approach to combat solid tumors in patients.

## MATERIALS AND METHODS

### Mice

C57BL/6J mice were purchased from Janvier and NOD/SCID/IL2Rγ^−/−^ mice from Charles River France. CMKLR1 knockout (CMKLR1^−/−^) mice were obtained from Deltagen and backcrossed into the C57BL/6J background for over 20 generations. The K5 chemerin mice expressing bioactive chemerin under control of the keratin K5 promoter were described and characterized elsewhere [40]. Mice were maintained in a specific pathogen-free environment and, except otherwise stated, were used between 6 and 10 weeks of age. In all experiments, animals were age-matched and distributed randomly into groups. All animal experiments were conducted in accordance with European guidelines and local regulations. They were approved by the local ethics committee (Commission d’Ethique du Bien-Etre Animal, CEBEA) of the ULB Medical School. All efforts were made to minimize suffering.

### Cell lines and human primary cells

Murine B16-F0 melanoma and Lewis lung carcinoma (LLC) cell lines were purchased from the American Type Culture Collection (ATCC). To produce cell lines expressing chemerin, B16 and LLC cells were transfected with pEFIN plasmids encoding a bioactive form of chemerin (1–157) not requiring proteolytic processing. Cells were grown in RPMI-1640 (Life Technologies) supplemented with 10% fetal bovine serum (Gibco), 1% sodium pyruvate (Gibco), 100 U/mL penicillin, and 100 μg/mL streptomycin (Invitrogen). The cells were cultured at 37°C in a humidified atmosphere containing 5% CO_2_. The various clones were regularly tested negative for mycoplasma infection. To assess the influence of chemerin on the growth of LLC and B16 cell lines *in vitro*, the cells were cultured in the presence of 100 nM mouse recombinant chemerin (R&D Systems, 2325-CM-025).

Human umbilical vein endothelial cells (HUVEC) were obtained from ScienCell Research Laboratories (#8000) or Lonza (C2517A) and cultured on fibronectin-coated flasks in endothelial cell basal medium (EBM-2; CC-3156) containing EGM-2 supplements (CC-4176). Human brain vascular pericytes (HBVP, ScienCell Research Laboratories, #1020) were cultured on poly-L-lysine-coated flasks (10 mg/ml, #0413) in pericyte medium (PM, #1201) containing 2% FBS, 1% pericyte growth supplement (PGS, #1252), 100 U/mL penicillin and 100 μg/mL streptomycin (Invitrogen). Human lung fibroblasts (NHLF, Lonza, CC-2512) were cultured in fibroblast growth basal medium (FBM, Lonza, CC-3131) containing FGM-2 supplements and growth factors (Lonza, CC-4126). All cell types were used between passages 2 and 6.

### Tumor models

B16 or LLC cells (10^6^) were inoculated subcutaneously into the back of syngeneic C57BL/6J mice. The size of the resulting tumors was monitored daily with a caliper, and the tumor volume was estimated by the formula *V* = (length × width^2^)/2. At the end of the experiment, the mice were killed by cervical dislocation, and the tumors were collected for further analysis.

Axitinib (Selleckchem, S1005) was dissolved in sterile water containing 0.5% hydroxy-propyl-methylcellulose (Sigma, M7140) and 0.1% Tween-80 (Sigma, P1754). Mice were randomly divided into a control group and a treatment group. Two days after the tumor cell graft, mice were treated twice a day by oral gavage with vehicle or axitinib (10 mg/kg) for a period of 5 days.

### Flow cytometry analyses

Tumors were cut into small fragments (about 1 mm^3^) and digested in HBSS medium containing 5% fetal bovine serum, 1 mg/mL collagenase D (Roche), and 200 U/mL DNAse I (Roche) for 1 h 30 at 37°C on a rocking plate. Collagenase D activity was blocked by the addition of 5 mM EDTA, and the cell suspension was rinsed with PBS and tissue debris removed by filtering through a 70-μm nylon mesh. Single-cell suspensions were incubated for 30 min at 4°C with anti-CD16/CD32 Fc Block (eBioscience, 14-0161-86) and a mixture of antibodies in FACS buffer (PBS containing 1% FCS, 1 mM EDTA and 0.1% NaN_3_). Flow cytometry analysis was performed on a LSRFortessa instrument (BD Biosciences) and analyzed using the FlowJo software. The antibodies used were directed to CD45 (47-0451 and 17-0451-83), NK1.1 (12-5941-82), CD3 (17-0032-82), CD4 (48-0041) from eBioscience, and CD8 (551162), CD11b (550993 and 553311), CD11c (550261) and Gr1 (552093) from BD Pharmingen.

### Histological procedures

Tumors were embedded in OCT and sections post-fixed in acetone for 10 minutes at room temperature. For immunofluorescence staining, the sections were blocked with 1% rat serum, then incubated for 2 h 30 at 4°C with PE-conjugated mouse anti-CD31 (12-0311-83) and APC-conjugated mouse anti-α-SMA (50-9760) from eBioscience. For the labeling of hypoxic tissue, pimonidazole HCl (Hypoxyprobe-1, Hypoxyprobe) was injected intravenously (60 mg/kg body weight) 90 minutes before sacrifice. OCT-embedded tumors were immunostained with a FITC-conjugated anti pimonidazole monoclonal (Mab1) following the manufacturer's instructions. Nuclei were stained with Hoechst 33342 (Molecular Probes, H3570, 1:4000), and slides were mounted in DAKO mounting medium supplemented with 2.5% 4-diazabicyclo[2.2.2]octane (DABCO, Sigma). Slides were counter-stained by hematoxylin-eosin and mounted in Entellan (Merck). Images were acquired using a Zeiss LSM 780 confocal microscope or a Zeiss AxioImager Z1 (Carl Zeiss). They were analyzed and their contrast adjusted with the ImageJ software.

### Angiogenesis assays

For the wound healing assay, HUVECs were seeded in Culture-Insert 2 Wells (Ibidi) at a density of 12,000 cells per insert in EGM-2 medium until confluence. After an additional 3 hours in serum-free medium, the insert was removed, leaving a 500 μm cell-free gap. Cell migration was assessed in EBM medium containing 0.5% FBS and supplemented or not with 5 nM recombinant human chemerin (R&D Systems). Images were captured at 24 and 48 h, using a ZOE Fluorescent Cell Imager (BioRad) at 20× magnification, and the cell-free area was measured in three fields using the ImageJ software.

Tube formation assays were performed as described previously [58]. Briefly, 96-well plates were precoated with Matrigel (50 μl/well, Corning, #356231), centrifuged at 300 g for 10 min at 4°C, and incubated at 37°C for 30 min to allow the matrix to solidify. Medium containing 12,000 HUVECs (monocultures), or 10,000 HUVECs and 2,000 pericytes (co-cultures) in 100 μl, supplemented or not with VEGF (Lonza, #CC-4114A) and/or 10, 20 and 50 nM human chemerin was added to each well, and the plates incubated in a humidified chamber at 37°C in a 5% CO_2_ incubator. The formation of capillary-like structures was monitored, photographed at 4, 6, 8, and 24 hours, using a Zeiss Axio observer Z1 microscope with a 2.5x objective. The images were processed using the Angiotool plugin in ImageJ or the Wimtube tool from Wimasis.

The bead sprouting assay was performed essentially as described [59]. Briefly, HUVECs (2 × 10^6^ cells) were mixed with 5000 Cytodex-3 beads (Sigma, #C2375) in 1 ml of EGM-2 complete medium and incubated for 4 h at 37°C in a 5% CO_2_ incubator with gentle mixing every 20 min. After incubation, the coated beads were transferred to a 25-cm^2^ tissue culture flask and left overnight in EGM-2 at 37°C and 5% CO_2_. The following day, the beads were resuspended in 15 ml of a 2 mg/ml fibrinogen solution containing 0.15 units/ml aprotinin, and 0.5 ml of this suspension was added to 0.625 units of thrombin (Sigma) in wells of a 24-well plate. Plates were incubated for 20 min at room temperature and for 1 h at 37°C and 5% CO_2_ to allow fibrin clotting. EGM-2 (1 ml) and 20,000 fibroblasts were added to each well. The concentration of growth factor was reduced by half from day 5, and the beads were monitored daily and imaged at days 7 and 10 on a Leica microscope (DMI6000B) with a 5x objective. For each frame, a Z-stack collection of 10 to 12 focus planes separated by 10 μm was recorded using the LAS X life science microscope software (Leica). The images were processed in ImageJ, and the surface and length of sprouts were quantified by the WimSprout software (Wimasis).

### RT-qPCR

B16 and LLC cells or minced tissues were lysed in TRIzol reagent (Life Technologies), and mRNAs were extracted using RNAeasy minikit (Qiagen). RNA samples (1 μg) were treated with DNase I (Invitrogen Life Technologies) and transcribed into cDNA using oligoDT and SuperscriptIII (Invitrogen Life Technologies). Reactions were performed in a 20 μl final volume using the Power SYBR Green PCR Master Mix (ThermoFisher) on an Applied Fast 7500 thermocycler. Transcripts were normalized to glyceraldehyde-3-phosphate dehydrogenase (GAPDH) transcripts abundance analyzed in parallel. Reverse and forward primers used were 5′-AAGCTCCAGCAGACCAACTG-3′ (forward) and 5′-TTTACCCTTGGGGTCCATTT-3′ (reverse) for chemerin, 5′-CCATGTGCAAGATCAGCAAC-3′ (forward) and 5′-GCAGGAAGACGCTGGTGTA-3′ (reverse) for CMKLR1, 5′-GCTGCTGCTTATGGGCTTCTC-3′ (forward) and 5′-TCACTGGGCAGTTTCTAGGAG-3′ (reverse) for GPR1 and 5′-AAGGGCTCATGACCACAGTC-3′ (forward) and 5′-CAGGGATGATGTTCTGGGCA-3′ (reverse) for GAPDH.

### RNA sequencing and transcriptome analysis

Tumor tissue was lysed in TRIzol reagent (Life Technologies). Total RNA was extracted with the RNAeasy micro kit (Qiagen) following the manufacturer’s instructions. RNA quality was checked using a Bioanalyzer 2100 (Agilent Technologies). Indexed cDNA libraries were obtained using the TruSeq RNA sample preparation kit (Illumina) following the manufacturer’s recommendations. The multiplexed libraries (10 pM) were loaded on flow cells, and sequences were produced using a HiSeq PE Cluster Kit v4 and TruSeq SBS Kit v3-HS from a HiSeq 1500 (Illumina) at the Brussels Interuniversity Genomics High Throughput core (http://www.brightcore.be/). Approximately 35 millions paired-end reads per sample were mapped against the mouse reference genome (GRCm38.p4/mm10) using the STAR software to generate read alignments for each sample. The annotation file Mus_musculus.GRCm38.84.gtf was obtained from the http://ftp.Ensembl.org server. Following transcript assembly, gene level counts were obtained using HTSeq. The data have been deposited in NCBI’s Gene Expression Omnibus and are accessible through GEO Series accession number GSE98672. Differential gene expression was calculated on the Degust website (http://degust.erc.monash.edu/) using EdgeR. Gene signatures were analyzed on the Gorilla (http://cbl-gorilla.cs.technion.ac.il) and GSEA (http://software.broadinstitute.org/gsea/index.jsp) websites [60, 61].

### Blood vessel permeability assay

The ears of K5-chemerin, CMKLR1^−/−^ and littermate control mice were treated daily for 3 days with 12-O-tetradecanoyl phorbol-13-acetate (TPA, 37.5 μM in acetone, Sigma) or the solvent alone. On the fourth day, mice were anesthetized with 4% isoflurane and received an i.p. injection of 1% Blue Evans (4 mg/kg, Sigma) in PBS. 24 hours later, mice were sacrificed, and the ears were collected and placed overnight in 200 μl of formamide at 65°C. After centrifugation, the optical density of the supernatant was measured at 630 nm.

### Statistical analyses

Statistical analyses and data graphing were performed using Prism 6 (GraphPad Software). Statistical significance was calculated by the Mann-Whitney test. *P*-values < 0.05 were considered significant.

## SUPPLEMENTARY MATERIALS



## Abbreviations

cAMP: cyclic adenosine monophosphate
CCRL2: CC-motif chemokine receptor-like 2
CHO: Chinese hamster ovary
CMKLR1: chemokine like receptor 1
CXCL, CXC: chemokine ligand
DC: dendritic cell
EC: endothelial cell
ERK: extracellular signal-regulated kinase
FDR: false discovery rate
FGF: fibroblast growth factor
HUVEC: human umbilical vein endothelial cell
IL2Rγ: interleukin 2 receptor gamma chain
K5: keratin 5
LLC: Lewis lung carcinoma
MDSC: myeloid-derived suppressor cell
MMP: matrix metalloproteinase
NK: natural killer cell
NOD: non-obese diabetic
PI3K: phosphoinositide 3-kinase
RARRES2: retinoic acid receptor responder 2
SPF: specific pathogen-free
VEGF: vascular endothelial growth factor
