# Supplementary material for: The antitumoral effects of chemerin are independent from leukocyte recruitment and mediated by inhibition of neoangiogenesis
Source: Oncotarget. 2021 Sep 14;12(19):1903–19. doi: 10.18632/oncotarget.28056 (PMC8448509; doi:10.18632/oncotarget.28056)
Supplement: Supplementary file 1 [file oncotarget-12-1903-s001.pdf]

The antitumoral effects of chemerin are independent from leukocyte recruitment and mediated by inhibition of neoangiogenesis

SUPPLEMENTARY MATERIALS

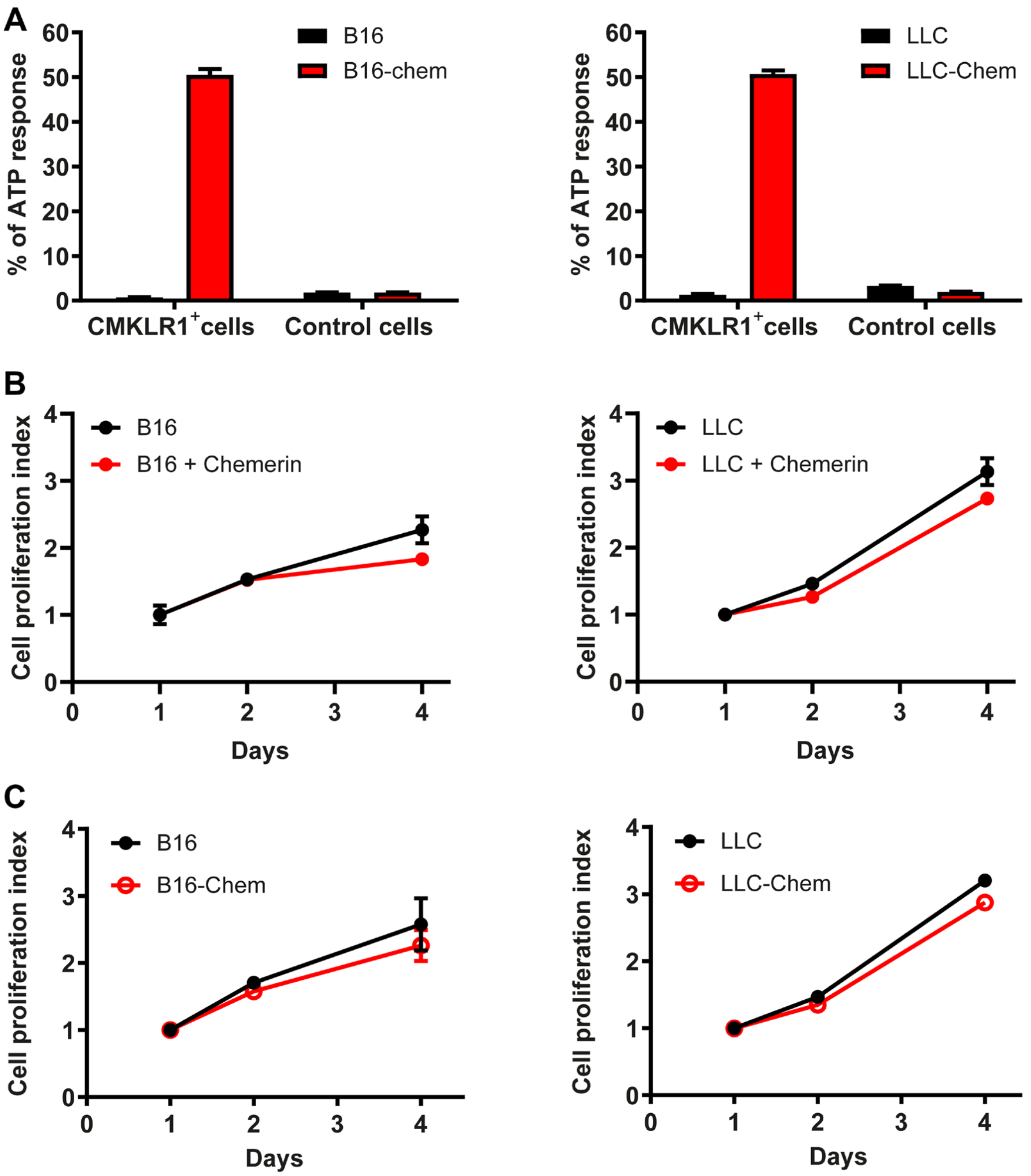

Supplementary Figure 1: Characterization of B16 and LLC cell lines. (**A**) The production of bioactive chemerin secreted by B16 and LLC cell lines was quantified in the culture medium by measuring its activity on a CHO-K1 cell line expressing CMKLR1 in an aequorin-based calcium mobilization assay. The data represent the luminescent signal obtained for 40 ml of conditioned medium, normalized to the response obtained for 10 µM ATP, used as a reference control. (**B**, **C**) Proliferation rate of LLC and B16 cell lines expressing or not chemerin (B) and of LLC and B16 cell lines in the presence or not of chemerin (100 nM) (C). The number of cells was counted on days 1, 2 and 4, and normalized according to day 1. The data (mean ± SEM) are representative of 2 independent experiments with 5 wells per condition.

Ensembl ID	Gene Name	Description	LLC 1	LLC 2	LLC-chem 1	LLC-chem 2	LLC (mean ± sem)	LLC-chem (mean ± sem)	LLC		LLC-chem	
ENSMUSG00000031871	Cdh5	Cadherin 5	51,4	34,1	9,9	5,3	43 ± 09	08 ± 02				
ENSMUSG00000039167	Adgrl4	Adhesion G protein-coupled receptor L4	12,6	5,8	3,3	1,0	09 ± 03	02 ± 01				
ENSMUSG00000045930	Clec14a	C-type lectin domain family 14, member a	10,1	9,3	2,4	2,0	10 ± 00	02 ± 00				
ENSMUSG00000039706	Ldb2	LIM domain binding 2	5,3	5,9	2,8	1,2	06 ± 00	02 ± 01				
ENSMUSG00000073599	Ecscr	Endothelial cell surface expressed chemotaxis and apoptosis regulator	11,6	9,1	3,6	2,2	10 ± 01	03 ± 01				
ENSMUSG00000046916	Myct1	Myc target 1	5,1	3,1	1,3	0,5	04 ± 01	01 ± 00				
ENSMUSG00000046768	Rhoj	Ras homolog gene family, member J	73,4	84,0	58,5	53,6	79 ± 05	56 ± 02				
ENSMUSG00000001930	Vwf	Von Willebrand factor homolog	19,3	13,9	5,8	5,5	17 ± 03	06 ± 00				
ENSMUSG00000033191	Tie1	Tyrosine kinase with immunoglobulin-like and EGF-like domains 1	25,6	15,3	5,1	2,2	20 ± 05	04 ± 01				
ENSMUSG00000062960	Kdr	Kinase insert domain protein receptor	35,2	33,2	9,1	6,4	34 ± 01	08 ± 01				
ENSMUSG00000001946	Esam	Endothelial cell-specific adhesion molecule	27,9	15,8	6,4	3,7	22 ± 06	05 ± 01				
ENSMUSG00000027435	Cd93	CD93 antigen	168,6	161,7	56,0	32,7	165 ± 03	44 ± 12				
ENSMUSG00000020154	Ptprb	Protein tyrosine phosphatase, receptor type, B	16,9	11,9	3,8	2,5	14 ± 03	03 ± 01				
ENSMUSG00000056492	Adgrf5	Adhesion G protein-coupled receptor F5	29,7	25,0	7,1	3,9	27 ± 02	05 ± 02				
ENSMUSG00000029309	Sparcl1	SPARC-like 1	63,2	35,4	15,7	9,5	49 ± 14	13 ± 03				
ENSMUSG00000054690	Emcn	Endomucin	14,8	9,2	3,6	1,7	12 ± 03	03 ± 01				
ENSMUSG00000032125	Robo4	Roundabout homolog 4	16,7	8,8	2,8	1,6	13 ± 04	02 ± 01				
ENSMUSG00000026814	Eng	Endoglin	116,5	115,9	112,9	108,1	116 ± 00	110 ± 02				
ENSMUSG00000006386	Tek/Tie2	Endothelial-specific receptor tyrosine kinase	25,1	53,2	17,1	11,3	39 ± 14	14 ± 03				
ENSMUSG00000045092	S1pr1	Sphingosine-1-phosphate receptor 1	32,9	28,9	23,7	15,0	31 ± 02	19 ± 04				
ENSMUSG00000030111	A2m	Alpha-2-macroglobulin	0,5	0,7	0,5	0,2	01 ± 00	00 ± 00				
ENSMUSG00000053062	Jam2	Junction adhesion molecule 2	6,1	7,4	6,4	5,3	07 ± 01	06 ± 01				
ENSMUSG00000005583	Mef2c	Myocyte enhancer factor 2C	20,8	21,9	15,2	9,3	21 ± 01	12 ± 03				
ENSMUSG00000028339	Col15a1	Collagen, type XV, alpha 1	116,9	77,6	30,9	14,9	97 ± 20	23 ± 08				
ENSMUSG00000020717	Pecam1	Platelet/endothelial cell adhesion molecule 1	71,2	46,2	18,5	9,4	59 ± 13	14 ± 05				
ENSMUSG00000059588	Calcrl	Calcitonin receptor-like	25,3	22,2	42,5	44,9	24 ± 02	44 ± 01				
ENSMUSG00000025784	Clec3b	C-type lectin domain family 3, member b	158,9	353,8	142,3	84,2	256 ± 97	113 ± 29				
ENSMUSG00000034845	Plvap	Plasmalemma vesicle associated protein	58,9	40,1	18,2	12,6	49 ± 09	15 ± 03				
ENSMUSG00000026678	Rgs5	Regulator of G-protein signaling 5	73,8	43,3	16,3	11,8	59 ± 15	14 ± 02				
ENSMUSG00000090958	Lrrc32	Leucine rich repeat containing 32	42,1	37,9	17,4	15,7	40 ± 02	17 ± 01				
ENSMUSG00000057098	Ebf1	Early B cell factor 1	25,7	48,2	34,9	35,9	37 ± 11	35 ± 01				
ENSMUSG00000022220	Adcy4	Adenylate cyclase 4	7,3	5,2	2,3	1,7	06 ± 01	02 ± 00				
ENSMUSG00000000530	Acvrl1	Activin A receptor, type II-like 1	38,5	51,6	20,4	14,8	45 ± 07	18 ± 03				
ENSMUSG00000031486	Adgra2	Adhesion G protein-coupled receptor A2	196,1	205,3	171,4	171,1	201 ± 05	171 ± 00				
ENSMUSG00000044338	Aplnr	Apelin receptor	33,4	18,5	5,9	4,3	26 ± 07	05 ± 01				
ENSMUSG00000032766	Gng11	Guanine nucleotide binding protein (G protein), gamma 11	18,1	21,6	19,2	17,2	20 ± 02	18 ± 01				
ENSMUSG00000044629	Cnrip1	Cannabinoid receptor interacting protein 1	15,9	18,1	18,1	18,3	17 ± 01	18 ± 00				
ENSMUSG00000064262	Gimap8	GTPase, IMAP family member 8	6,0	3,9	1,1	0,9	05 ± 01	01 ± 00				

2	
1	
0	
-1	
-2	

Supplementary Figure 2: Angiogenesis signature in LLC tumors expressing or not chemerin. The expression data were extracted from our RNAseq results performed on LLC tumors expressing (LLC-chem) or not chemerin for the first 40 genes of an angiogenesis signature described for a set of human cancer types (Masiero et al., 2013). Two genes had no orthologs in the mouse genome. The number of reads per million (RPM) is given for the replicates in each group, as well as the mean ± SEM, and the color-coded variation relative to the overall mean, in a log2 scale.

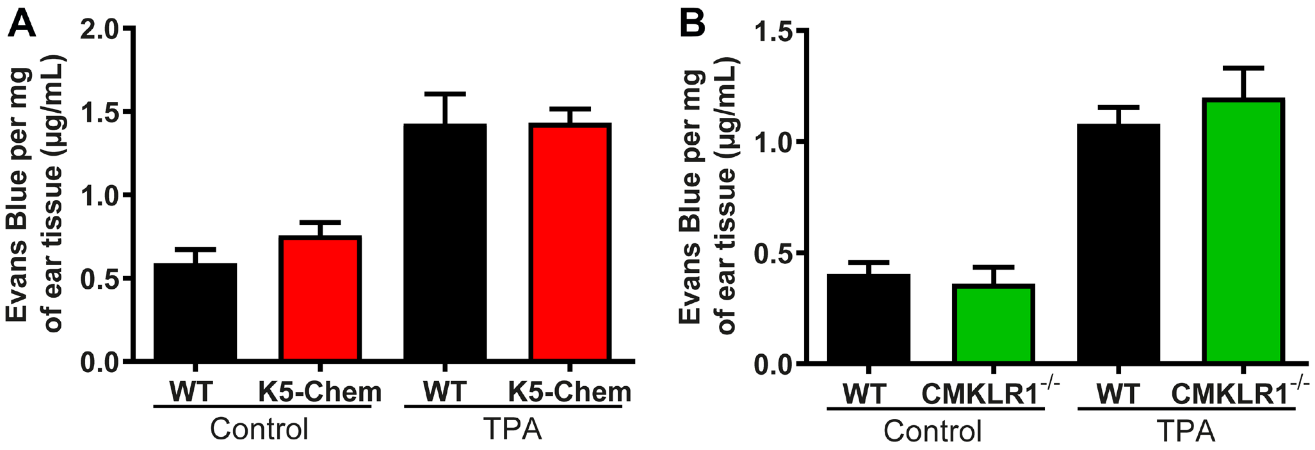

Supplementary Figure 3: Chemerin expression or CMKLR1 invalidation does not affect vascular permeability in mice. Ears of K5-chemerin (**A**) or CMKLR1$^{-/-}$ (**B**) mice and their controls were treated or not by TPA painting (37.5 µM). Four days later, Evans Blue (4 mg/kg) was injected i.p. and the mice were sacrificed 24 h later. Evans Blue extravasation was quantified by measuring the optical density at 630 nm in the tissue homogenate following centrifugation. The data combine two independent experiments with 5 mice per condition in each experiment.
